# Evaluation of Salt Resistance of Six Apple Rootstocks

**DOI:** 10.3390/ijms252312568

**Published:** 2024-11-22

**Authors:** Lun Li, Guolin Chen, Qingrong Sun, Qing Wang, Sen Wang, Haibo Wang, Zhihua Ni, Caina Jiang, Linguang Li, Tianhong Li

**Affiliations:** 1 Frontiers Science Center for Molecular Design Breeding, College of Horticulture, China Agricultural University, Beijing 100193, China; 18508436194@163.com (L.L.); jcaina@163.com (C.J.); 2 Shandong Institute of Pomology, Taian 271000, China; sdipss@163.com (Q.S.); wangs6645@163.com (S.W.); wanghaibo992@126.com (H.W.); 3 College of Horticulture and Engineering, Shandong Agricultural University, Taian 271018, China; cgl19981222@163.com (G.C.); w19806103452@163.com (Q.W.); 4 College of Horticulture, Jilin Agricultural University, Changchun 130118, China; nizhihua0331@163.com

**Keywords:** fruit tree rootstock, gene expression, physiological indicators, salt stress

## Abstract

Apples (*Malus domestica Borkh)* are important fruits in China; however, salt stress is severe in northern regions, and the key to plant resistance to salt stress lies in the rootstock. Therefore, it is necessary to explore rootstocks with strong salt resistance for the development of the apple industry. This study used tissue culture seedlings of six apple rootstocks, namely, ‘71-3-150’, ‘54-118’, ‘M9T337’, ‘GM256’, ‘ML176’, and ‘ML2’, as experimental materials. The seedlings were treated with a medium containing 150 mM NaCl, and the physiological indicators and related gene expression responses of several rootstocks were studied after salt stress. The results showed that salt stress affects the growth of both the aboveground and underground parts of plants. Through physiological indicators and the related gene expression responses of rootstocks, it was observed that salt stress significantly increased Na^+^ contents in different rootstocks. Simultaneously, the activity of various antioxidant enzymes and the expression levels of related genes also increased. In summary, by analyzing the parameters of various physiological indicators, it can be concluded that among the studied rootstocks, the ‘71-3-150’ and ‘54-118’ rootstocks have the strongest resistance to salt stress, while the ‘M9T337’ and ‘GM256’ rootstocks exhibit moderate resistance, and the ‘ML176’ and ‘ML2’ rootstocks have the weakest resistance.

## 1. Introduction

Apples are a widely cultivated fruit, and they contain a variety of vitamins and are rich in nutritional value. In recent years, with the continuous increase in apple planting area and yields, great contributions have been made to accelerating the development of the rural economy in China, encouraging farmers to increase production and income, and improving farmers’ income levels [1]. The apple industry is an important part of China’s agricultural economy and plays a key role in the adjustment of the overall agricultural structure and the increase in farmers’ income [2]. At the same time, the highly salt resistant apple rootstock has laid a foundation for the development and utilization of high-quality germplasm resources in China in the future [3]. High concentrations of salt disrupt the balance within plant bodies, resulting in water scarcity. This not only affects the yield of apples but also inflicts significant damage on fruit quality [4]. China is the largest producer of apples, but with the increasing global climate change, salt stress in the soil seriously affects the yield and quality of apples. Long-term salt stress has also become a prominent limiting factor in many apple-producing areas [5]. Research has shown that apple trees typically use grafting techniques to graft scions onto resistant rootstocks, thereby improving the quality of apples [6]. In order to better adapt to salt stress, selecting rootstocks with strong salt resistance and adaptability to salinization is essential to fundamentally address the problem [7]. However, the salt tolerance of different rootstocks varies greatly, and most rootstocks only retain moderate resistance. Therefore, the evaluation of the salt tolerance of different rootstocks is particularly important.

There have been many studies on the effects of salt stress. Salt stress can affect the growth of plant leaves and the growth status of roots to varying degrees [8]. Simultaneously, salt stress can affect the growth and development of plants, in addition to morphological changes [9,10]. Salt stress not only impacts the growth of aboveground parts of plants but also transmits stress signals to the roots. The research consensus is that morphological indicators, such as plant height and fresh weight, will change after being affected by salt stress [11]. After root damage, salt stress can affect indicators such as root length and biomass, reducing root vitality. In addition, various physiological indicators of plants will also change under the influence of salt stress. The increase in Na^+^ content and decrease in K^+^ content in the plant body cause ion stress, resulting in metabolic disorders, the accumulation of various toxic substances in the plant body, and changes in membrane permeability, further affecting the normal growth of plants [12]. The osmotic substances in plants can also be affected by salt stress. Plants regulate their growth by accumulating osmotic substances, such as proline, soluble sugars, and soluble proteins, in order to alleviate stress [13]. Plant ion transporters are activated under salt stress to regulate ion homeostasis [14]. Salt stress can also result in the accumulation of reactive oxygen species in plants, such as increased levels of superoxide anions and hydrogen peroxide [15]. In order to eliminate the impact of reactive oxygen species on plants, the levels of superoxide dismutase (SOD), peroxidase (POD), and catalase (CAT) in the plant body undergo certain changes under salt stress, thereby reducing the harm caused by reactive oxygen species in plants [16]. The endogenous hormone signaling pathway in plants also participates in regulating the salt stress response, and the levels of Gibberellin 4 (GA_4)_ and salicylic acid (SA) in plants decrease under the influence of salt stress [17]. The content of ABA increases, thereby improving the plant’s tolerance to salt stress [15]. Salt stress induces the expression of stress-signal-transduction-related genes [18,19]. Studies have shown that genes such as *MdSOS1* and *MdSOS2* play a role in salt stress, providing rootstocks with better adaptability to salt stress.

Different rootstock types exhibit different resistance; therefore, the type of rootstock directly affects the resistance of apple fruit trees. However, commonly used rootstocks in apple production are cold resistant and drought-resistant [20]. There is relatively little research on the advantages of salt resistance, and it is still of great significance to supplement the evaluation of rootstocks that have good adaptability to salt stress.

This study compares the physiological responses of six apple rootstock varieties to salt stress and evaluates their tolerance to salt stress; this has important reference significance for improving fruit quality through grafting techniques in the future.

## 2. Results and Analysis

### 2.1. Changes in Apple Rootstock Plants Under Salt Stress

**Figure 1:**
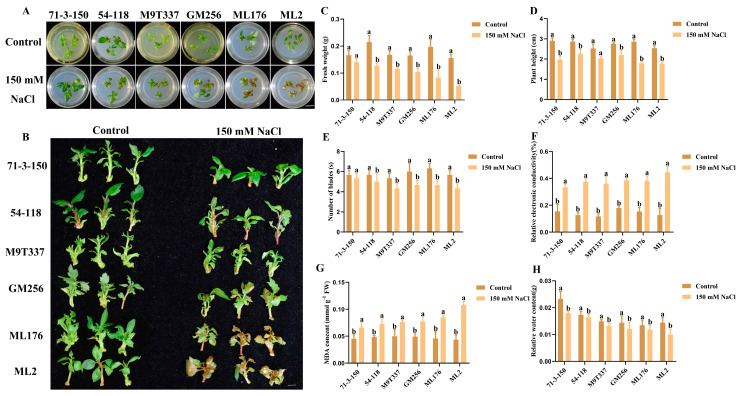
Changes in phenotype and physiological indexes of apple rootstock under salt stress. (**A**,**B**) Plant growth phenotype. Bar scale = 0.5 cm. (**C**) Fresh weight. (**D**) Plant height. (**E**) Number of blades. (**F**) Relative conductivity. (**G**) MDA content. (**H**) Relative water content. a and b indicate that the mean values of the control group and the salt stress treatment group are significantly different. Three separate experiments all had similar results. Data represent means ±SD.

The changes in plant growth indicators are the most direct indicators reflecting the response to salt stress. Under salt stress, the growth phenotypes of six apple rootstocks were observed (Figure 1A,B). The fresh weight, plant height, number of leaves, and relative water content of the six apple rootstocks exhibited a decreasing trend compared to the control and treatment groups (Figure 1C,D,F,H), while relative conductivity and MDA content exhibited an increasing trend (Figure 1E,G). Under the control conditions, the six rootstocks grew normally without any abnormalities. Under salt stress, the growth of ‘71-3-150’ and ‘54-118’ was the best, and their fresh weight and plant height were superior to several other rootstock types under salt stress; ‘M9T337’ and ‘GM256’ were relatively stable and in an intermediate state. The plant height, fresh weight, number of leaves, and relative water content of ‘ML176’ and ‘ML2’ were significantly lower than those of other rootstocks. The results showed that under salt stress, the aboveground parts of rootstocks ‘71-3-150’ and ‘54-118’ exhibited the best performance; ‘M9T337’ and ‘GM256’ were intermediately damaged, with a small number of leaves wilting and turning yellow, and some degree of leaf shedding was observed. The damage inflicted on ‘ML176’ and ‘ML2’ was severe, with most leaves turning yellow and shedding. Based on phenotypic symptoms and aboveground physiological indicators, the salt tolerance of six genotypes can be preliminarily evaluated as follows: ‘71-3-150’ and ‘54-118’ are highly salt-resistant, ‘M9T337’ and ‘GM256’ are intermediately salt-resistant, and ‘ML176’ and ‘ML2’ exhibit low salt resistance.

### 2.2. Changes in Apple Rootstock Leaves Under Salt Stress

**Figure 2:**
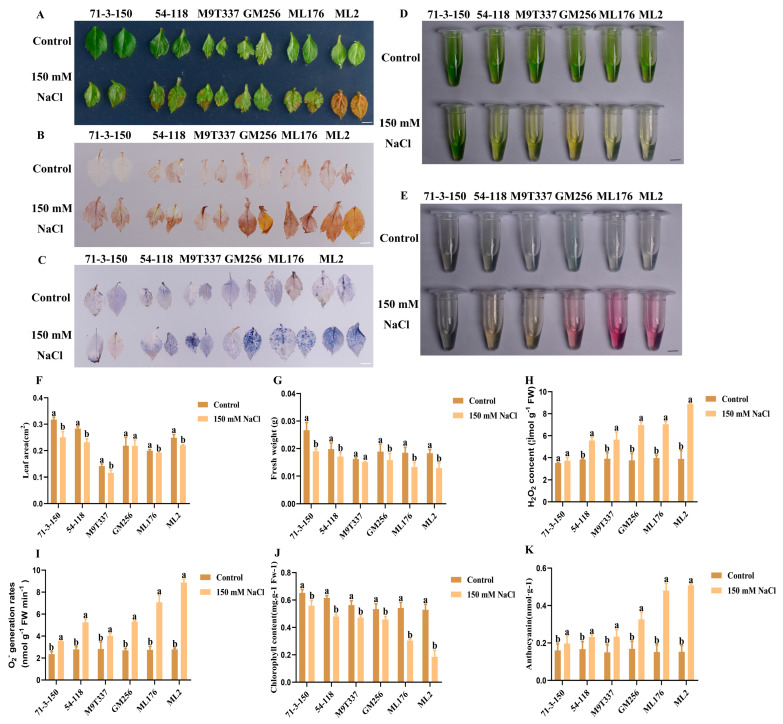
Changes in leaf phenotype and physiological indices of apple rootstock under salt stress. (**A**) Phenotype of plant leaves under salt stress. (**B**) Leaf DAB staining. (**C**) Leaf NBT staining. (**D**) Phenotype of chlorophyll content in plant leaves under salt stress. Bar scale = 0.5 cm. (**E**) Table of anthocyanin content in plant leaves under salt stress. Bar scale = 0.5 cm. (**F**) Leaf area. (**G**) Leaf fresh weight. (**H**) Hydrogen peroxide content. (**I**) Superoxide anion content. (**J**) Chlorophyll content. (**K**) Anthocyanin content. a and b indicate that the mean values of the control group and the salt stress treatment group are significantly different. Three separate experiments all had similar results. Data represent means ±SD.

The growth of plant leaves is one of the important indicators reflecting their survival under salt stress. Figure 2A shows the leaf phenotypes of six rootstocks. The growth status of the control group was good and uniform; under salt stress, the leaf area and fresh weight of plants were affected to varying degrees (Figure 2F,G). In this experiment, DAB staining and NBT staining were performed on the leaves (Figure 2B,C) to examine the changes in hydrogen peroxide and superoxide anion content (Figure 2H,I). The control group of six rootstocks exhibited transparent leaves after DAB staining and NBT staining; under salt stress, the leaves of the ‘71-3-150’ and ‘54-118’ rootstocks were slightly colored. The color on the leaves of the ‘M9T337’ and ‘GM256’ rootstocks was more pronounced, with almost all leaves of the ‘ML176’ and ‘ML2’ rootstocks stained. Simultaneously, changes were observed in the chlorophyll and anthocyanin contents of six rootstocks under salt stress (Figure 2D,E). The rootstocks grown under normal conditions had abundant chlorophyll and low anthocyanin contents, and they remained in a stable state. Under salt stress (Figure 2J), the ‘71-3-150’ and ‘54-118’ rootstocks had the highest chlorophyll content compared to other rootstocks under salt stress. The chlorophyll content in the ‘M9T337’ and ‘GM256’ rootstocks gradually decreased compared to the control group, while the chlorophyll content in the ‘ML176’ and ‘ML2’ rootstocks was the lowest and exhibited a significant decreasing trend. However, the contents of anthocyanins in the ‘71-3-150’, ‘54-118’, and ‘M9T337’ rootstocks were relatively low, while the content of anthocyanins in ‘GM256’ gradually increased. The content of anthocyanins in the ‘ML176’ and ‘ML2’ rootstock varieties significantly increased compared to the control group and other rootstock varieties. In summary, plants affected by salt stress indicated that ‘71-3-150’ and ‘54-118’ were least affected by salt stress, while the ‘ML176’ and ‘ML2’ rootstock varieties were most affected.

### 2.3. Changes in Leaf Structure of Apple Rootstocks Under Salt Stress

**Figure 3:**
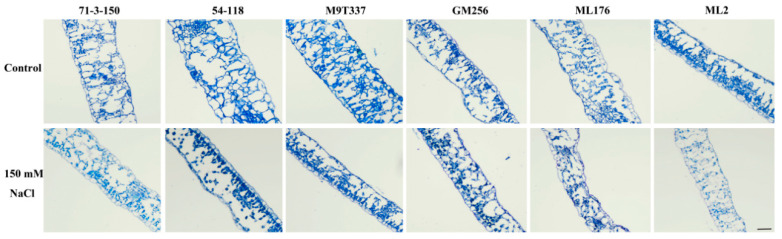
Changes in leaf structure of apple rootstock under salt stress. Bar scale = 500 μm.

The leaf anatomical structures of six apple rootstocks were observed under an optical microscope (Figure 3). The results showed that the leaf structure of the six rootstock control groups was intact and basically undamaged. Under salt stress, the leaf tissues of all six rootstocks decreased in size. Among them, the ‘71-3-150’ rootstock variety showed less damage compared to the control group, with abundant cell content and intact tissue parts. Compared with ‘71-3-150’, the palisade tissue distribution of the ‘54-118’, ‘M9T337’, and ‘GM256’ rootstock varieties was uneven, and it did not exhibit a complete state. The leaf structure of the ‘ML176’ and ‘ML2’ rootstock varieties showed significant damage, with loose cells, incomplete palisade tissues, and fractures compared to the control group. The results showed that under salt stress treatment, the leaf structure of all rootstocks decreased in clarity and varied in shape compared to the control group. Among them, the cell structure of the ‘71-3-150’ rootstock variety was the most complete, indicating that ‘71-3-150’ was least affected by salt stress and exhibited the strongest resistance.

### 2.4. Changes in Elements in Apple Rootstock Leaves Under Salt Stress

**Figure 4:**
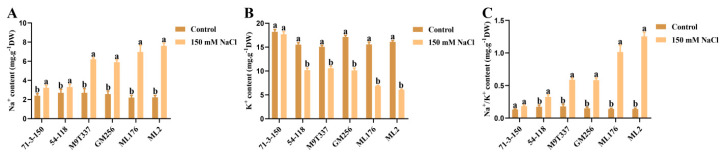
Changes in the hormones of apple rootstock under salt stress. (**A**) Na^+^ content. (**B**) K^+^ content. (**C**) Na^+^/K^+^ content. a and b indicate that the mean values of the control group and the salt stress treatment group are significantly different. Three separate experiments all had similar results. Data represent means ±SD.

Through experiments on salt stress treatment, the ion content in six genotypes of apple rootstocks was determined (Figure 4A,B). It can be observed in the figures that under control conditions, the Na^+^ content in all six apple rootstocks was consistent. Under salt stress, the Na^+^ content in the ‘71-3-150’ and ‘54-118’ rootstocks increased less compared to the control group, while the Na^+^ content in ‘M9T337’ and ‘GM256’ rootstocks significantly increased, and ‘ML176’ and ‘ML2’ had the highest Na^+^ content. The changes in K^+^ content in several apple rootstocks were not significant, while after salt stress treatment, the K^+^ content in several rootstocks decreased. Among them, the K^+^ content of the ‘71-3-150’ rootstock was basically consistent with the control group, while the K^+^ content in the ‘54-118’, ‘M9T337’, and ‘GM256’ rootstocks gradually decreased, and the K^+^ content in the ‘ML176’ and ‘ML2’ rootstocks significantly decreased. The results showed that under salt stress, the increase in the sodium ion content of ‘71-3-150’ and ‘54-118’ was significantly smaller than that of other rootstock varieties, and the sodium ion content in their tissues was lower. The potassium ion content in ‘71-3-150’ remained stable, while the potassium ion content in ‘54-118’, ‘M9T337’, and ‘GM256’ decreased significantly. ‘ML176’ and ‘ML2’ had the lowest potassium ion content among all the rootstock varieties studied.

### 2.5. Changes in Antioxidant Enzyme Activity and Osmotic Substances in Apple Rootstock Leaves Under Salt Stress

**Figure 5:**
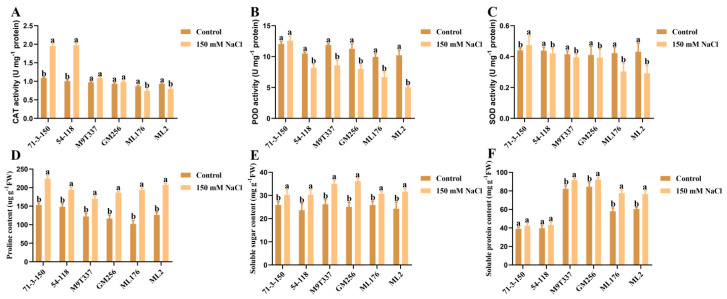
Changes in antioxidant enzyme activity and osmotic substances in apple rootstock leaves under salt stress. (**A**) CAT activity. (**B**) POD activity. (**C**) SOD activity. (**D**) Proline content. (**E**) Soluble sugar content. (**F**) Soluble protein content. a and b indicate that the mean values of the control group and the salt stress treatment group are significantly different. Three separate experiments all had similar results. Data represent means ±SD.

The antioxidant enzyme activity and osmotic substances in plant leaves can partially reflect the plant’s tolerance to salt stress (Figure 5). Under normal conditions, the antioxidant levels of the six rootstocks varied, which may be due to differences in genotype among the rootstocks. Under salt stress, the activities of CAT, POD, and SOD in the ‘71-3-150’ rootstock were consistent with those in the control group, while the activities of CAT, POD, and SOD in the ‘54-118’, ‘M9T337’, ‘GM256’, and ‘ML176’ rootstocks showed a decreasing trend (Figure 5A–C). The results showed that the ‘71-3-150’ rootstock variety had strong antioxidant-enzyme-scavenging functions after being affected by salt stress, thus maintaining the stable state of its active substances. At the same time, osmotic substances in various rootstock varieties were detected. Under salt stress, except for the ‘71-3-150’ and ‘54-118’ rootstock varieties, the levels of proline, soluble sugar, and soluble protein increased, and the changes were inconsistent (Figure 5D–F). This may be due to the fact that plants need to consume some nutrients in their bodies after being subjected to salt stress, and they need to mobilize these nutrients to adapt to the external salt stress environment. However, the osmotic substances in the ‘71-3-150’ and ‘54-118’ rootstock varieties remained relatively low, indicating that their rootstock varieties have resistance. Their osmotic regulation ability is strong, and they have the function of regulating the environment; thus, they do not require too much nutrition to resist the effects of adversity.

### 2.6. Changes in Plant Hormones in Apple Rootstocks Under Salt Stress

**Figure 6:**
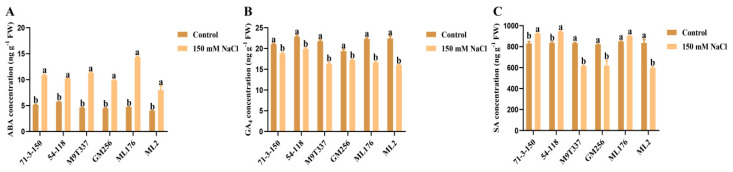
Changes in plant hormones in apple rootstocks under salt stress. (**A**) ABA content. (**B**) GA_4_ content. (**C**) SA content. a and b indicate that the mean values of the control group and the salt stress treatment group are significantly different. Three separate experiments all had similar results. Data represent means ±SD.

The changes in various hormones (ABA, GA_4_, and SA) in apple rootstock are important indicators of plant adaptability to salt stress conditions. The changes in hormones ABA, GA_4_, and SA in plants are shown in Figure 6A–C. Several plant hormones in the control group of six rootstocks tended to stabilize; after salt stress treatment, the plant hormones in several rootstocks showed different change trends. The ABA content was significantly higher than that in the control group (Figure 6A), while GA_4_ and SA plant hormones showed a decreasing trend (Figure 6B,C). After salt stress treatment, the ABA content in the ‘71-3-150’ and ‘54-118’ rootstocks exhibited little change compared to the control group. The ABA content in ‘M9T337’ and ‘GM256’ gradually increased, while the ABA content in the ‘ML176’ and ‘ML2’ rootstocks significantly increased. The GA_4_ content in the plant gradually decreased, while the hormone content in the ‘71-3-150’ and ‘54-118’ rootstocks remained most stable under salt stress. The hormone content in the ‘M9T337’ and ‘GM256’ rootstocks exhibited a decreasing trend, with ‘ML176’ and ‘ML2’ exhibiting the most significant decrease. Under salt stress, the SA content in plants exhibited a decreasing trend. The SA content in ‘71-3-150’, ‘54-118’, and ‘M9T337’ exhibited little change compared to the control group. The SA content in the ‘GM256’, ‘ML176’, and ‘ML2’ rootstocks gradually decreased, with the most significant decrease in ‘ML2’. The results showed that the ‘71-3-150’ and ‘54-118’ rootstock varieties had the strongest adaptability to salt stress.

### 2.7. Expression of Stress-Related Genes in Apple Rootstocks Under Salt Stress

**Figure 7:**
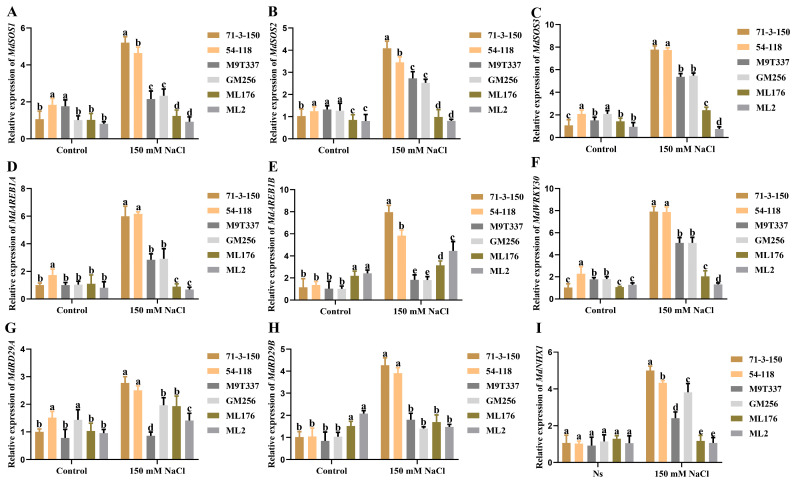
Expression of related apple rootstock leaf genes under salt stress. (**A**) Expression of *MdSOS1*. (**B**) Expression of *MdSOS2*. (**C**) Expression of *MdSOS3*. (**D**) Expression of *MdAREB1A*. (**E**) Expression of *MdAREB1B*. (**F**) Expression of *MdWRKY30*. (**G**) Expression of *MdRD29A*. (**H**) Expression of *MdRD29B*. (**I**) Expression of *MdNHX1*. a–e indicated differences in average gene expression between the control group and the salt stress treatment group. Three separate experiments had similar results.

Salt stress induces the expression of stress-related genes (such as SOS and AREB genes) to promote various physiological and biochemical responses in plants. The expression levels of stress-related genes are shown in Figure 7. It was found that there were differences in the expression levels of six rootstock-related genes under normal conditions, while the expression levels of related genes were significantly upregulated and varied under salt stress. Under salt stress, the expression levels of the *MdSOS1*, *MdSOS2*, *MdSOS3*, *MdAREB1A*, *MdAREB1B*, *MdWRKY30*, *MdRD29A*, *MdRD29B*, and *MdNHX1* genes were significantly increased and upregulated in rootstock varieties ‘71-3-150’ and ‘54-118’ (Figure 7A–I). The expression of *MdSOS2*, *MdSOS3*, *MdWRKY30*, and *MdNHX1* genes was significantly upregulated in ‘M9T337’ and ‘GM256’ (Figure 7B,C,F,I), while the upregulation of *MdSOS1*, *MdAREB1A*, and *MdAREB1B* was not as significant (Figure 7A,D,E). The expression levels of *MdAREB1B* and *MdRD29A* in ‘ML176’ and ‘ML2’ were slightly upregulated compared to the control group (Figure 7E,G). The transcription levels of *MdSOS1* and other genes in ‘71-3-150’ and ‘54-118’ were higher than those in other rootstocks under salt stress; this indicates that these two rootstocks can better induce the expression of related genes under salt stress, thereby reducing the harm of salt stress.

### 2.8. The Effect of Salt Stress on the Physiological Indicators of Rooting Rootstocks

**Figure 8:**
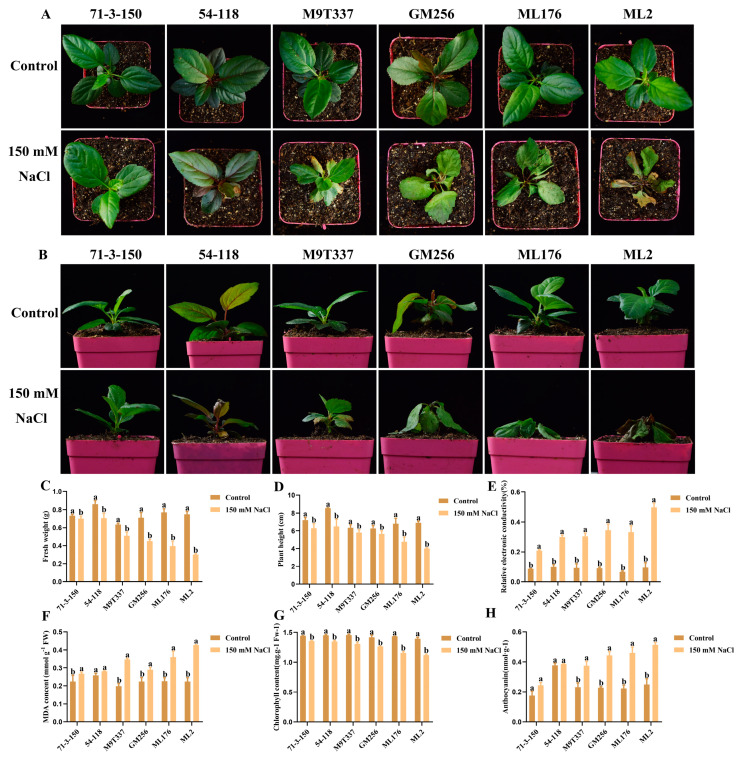
The effect of salt stress on physiological indicators of rooting rootstocks. Bar scale = 0.5 cm. (**A**,**B**) Plant growth phenotype under salt stress. (**C**) Fresh weight. (**D**) Plant height. (**E**) Relative conductivity. (**F**) MDA content. (**G**) Chlorophyll content. (**H**) Anthocyanin content. a and b indicate that the mean values of the control group and the salt stress treatment group are significantly different. Three separate experiments all had similar results. Data represent means ±SD.

In addition, we also regularly observed the growth of six rootstocks that survived after rooting. As shown in Figure 8A,B, under control conditions, the growth of the six rootstocks was uniform and neat. However, after salt stress treatment, the growth of ‘71-3-150’ and ‘54-118’ was relatively consistent, and they were less affected by salt stress. According to the phenotype, ‘M9T337’ and ‘GM256’ were affected by salt stress, and their leaves were damaged and wilted to varying degrees. ‘ML176’ and ‘ML2’ were the most severely damaged, with almost all leaves turning yellow, wilting, and falling off, which was significantly different from the others. The growth of rootstocks in the later stage was positively correlated with their adaptability to salt stress. Subsequently, it was observed that the fresh weight, plant height, and chlorophyll content of the rootstocks showed a decreasing trend under salt stress treatment, while the MDA, relative conductivity, and anthocyanin content of the rootstocks showed an increasing trend. However, overall, the physiological indicators of ‘71-3-150’ and ‘54-118’ exhibited significant advantages over other rootstocks, and ‘M9T337’ and ‘GM256’ were superior to ‘ML176’ and ‘ML2’.

### 2.9. The Effect of Salt Stress on the Rootstock Root System

**Figure 9:**
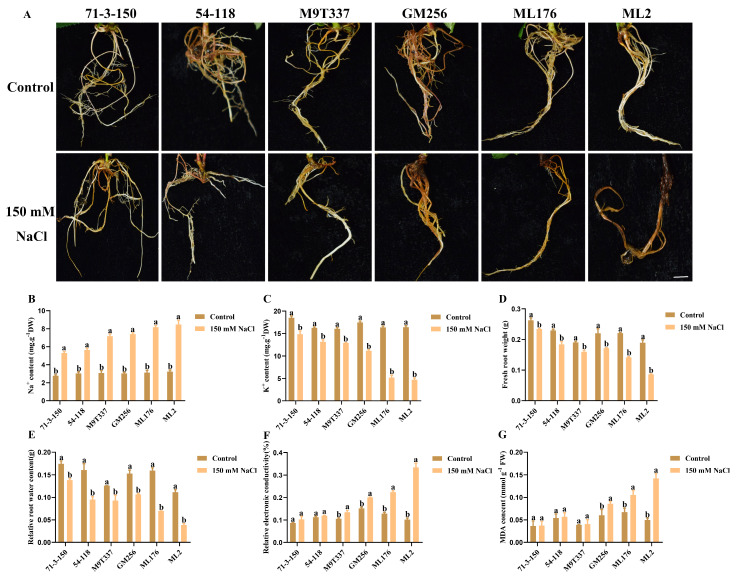
The effect of salt stress on the rootstock root system. Bar scale = 0.5 cm. (**A**) Root phenotype of rootstock. (**B**) Na^+^ content. (**C**) K^+^ content. (**D**) Fresh root weight. (**E**) Relative root water content. (**F**) Relative conductivity of roots. (**G**) MDA content in roots. a and b indicate that the mean values of the control group and the salt stress treatment group are significantly different. Three separate experiments all had similar results. Data represent means ±SD.

In this study, the Na^+^ content in all apple rootstock leaves significantly increased, indicating that salt stress disrupted the ion homeostasis in the rootstock, resulting in ion stress in the plants as shown on Figure 9A. However, the accumulation of Na^+^ in the roots of the rootstock was higher compared to the leaves, while the content of K^+^ exhibited a decreasing trend, similar to the leaves. In the control group, the root growth of rootstocks was uniform and consistent as shown on Figure 9B,C. However, under salt stress, rootstocks with low resistance and a high degree of decay were more affected by salt stress than rootstocks with intermediate resistance. The determination of indicators also proved that under salt stress, the fresh weight and water content of the roots of rootstocks ‘71-3-150’ and ‘54-118’ were higher than those of other rootstocks. The water content and fresh weight of the ‘M9T337’ and ‘GM256’ roots gradually decreased, while those of ‘ML176’ and ‘ML2’ were significantly reduced. After being affected by salt stress, the conductivity and MDA content of the ‘71-3-150’ and ‘54-118’ rootstocks increased slowly compared to other rootstocks, while the conductivity and MDA content in the roots of ‘ML176’ and ‘ML2’ rootstocks were the highest. Salt stress inhibited the vitality of rootstock roots by limiting their growth, while ‘71-3-150’ and ‘54-118’ both exhibited good trends.

## 3. Discussion

Salt stress has a certain impact on the growth and development of aboveground plant parts and root systems. At the same time, it can also cause various changes in enzyme activity and the leaf tissue within plants, and in severe cases, plants may even wither and die [21]. Moreover, the salt resistance of apples was closely related to the rootstock [22]. According to the tolerance of apple rootstocks to salt stress, they can be divided into high-resistance rootstocks, medium-resistance rootstocks, and low-resistance rootstocks. In this study, we mainly characterized the adaptability of different rootstocks to salt stress, which was mainly manifested in the differences in various physiological indicators of apple rootstocks. This experimental study proves that there are differences in the increasing and decreasing trends in various growth indicators of different rootstocks. Among them, the rootstocks ‘ML176’ and ‘ML2’ with poor salt resistance became weaker under salt stress, and in severe cases, their leaves fell off, withered, or even died, severely affecting the quality of the rootstocks (Figure 1 and Figure 2). The highly resistant apple rootstock performed significantly better than other apple rootstocks; it is worth exploring its physiological salt-resistance mechanism, which may play a key role in the future utilization and production of germplasm resources.

In this study, we observed that under the influence of salt stress, ion imbalance or ion toxicity in rootstocks inhibited the absorption of K^+^ by plants [23,24]. Under the influence of salt stress, the high accumulation of Na^+^ not only had toxic effects on plant ions but also resulted in osmotic stress, causing oxidative damage to cell membranes, resulting in plant dehydration and death, and severely affecting plant growth and development [5]. At the same time, with the increase in Na^+^ content in plant cells, the chlorophyll contents of plants exhibit strong sensitivity to salt stress [25]. Similarly, in this study, the chlorophyll content of plants was positively correlated with the salt and alkali tolerance of apple rootstocks. The chlorophyll content of highly salt-resistant rootstocks ‘71-3-150’ and ‘54-118’ remained stable compared to the control (Figure 2). We observed that rootstocks severely affected by salt stress had higher levels of leaf damage, which may be related to chloroplast synthesis and photosynthesis (Figure 3). In addition, studies have shown that rootstocks with poor salt tolerance have higher anthocyanin contents under salt stress [26], and this is consistent with our research results. High-resistance rootstocks may resist the effects of salt stress by adjusting ion balance while maintaining ion homeostasis to endow them with salt-tolerant characteristics [27]. Excessive accumulation of ROS can result in cell membrane damage, and the relative conductivity and MDA accumulation of plants are closely related to the accumulation of ROS (Figure 1F; Figure 2H,I). The higher the degree of enzyme and cell membrane peroxidation in plant cells, the higher the content of malondialdehyde (MDA) and relative conductivity in the plant body [28]. In this study, high-resistance rootstocks exhibited certain ROS scavenging abilities. The ROS scavenging ability of low-salt-resistance rootstocks ‘ML176’ and ‘ML2’ was weak (Figure 1F,G), and plants resist the negative effects of salt stress by improving their osmotic substances [29]. Proline, soluble sugars, and soluble proteins are important osmotic substances that reflect the effects of salt stress [30]. In this study, rootstocks adapted to salt stress by regulating their own osmotic substances and forming small molecule compounds (Figure 5). The mechanism by which rootstocks of different genotypes relieve osmotic stress is not yet clear, and further research is needed. During osmotic stress, plants accumulate a certain amount of ROS [31]. The growth and metabolism of apple rootstocks are also affected by salt stress, and plants themselves produce antioxidant enzymes to eliminate the impact of ROS on their tissues [32,33]. Under normal growth conditions, the presence of antioxidant enzymes and ROS in plants is in a state of equilibrium, which can be disrupted under stress conditions [34], thereby affecting and damaging the activity of cell membranes and enzymes. Due to the differences in the genotypes of different rootstocks, the accumulated antioxidant enzymes, such as superoxide dismutase (SOD), peroxidase (POD), and catalase (CAT) in rootstocks, are not consistent. In this study, the genotypes of the six rootstocks were different; thus, their nutrient indicators changed differently under salt stress. The high-salt-resistance apple rootstocks ‘71-3-150’ and ‘54-118’ had stable nutrients and could actively resist the effects of salt stress. These key enzymes play a crucial role in clearing the hazards caused by reactive oxygen species [15]. DAB and NBT staining methods are commonly used to study H_2_O_2_ and O_2_ contents in plants [35]. The deeper staining of leaves in the treatment group of low-salt-resistance rootstock indicates the higher accumulation of H_2_O_2_ and O_2_ contents (Figure 2). Simultaneously, after salt stress, *MdSOS1*, *MdSOS2*, *MdSOS3*, *MdNHX1*, and *MdWRKY30* exhibited a significant upregulation trend in the roots and leaves of plants [18,36]. Under salt stress, it is necessary to induce the expression of stress-signal-transduction-related genes and physiological and biochemical reactions in plants [37]. *MdAREB1A* and *MdAREB1B* genes are expressed under drought stress [38], and *MdRD29A* and *MdRD29B* genes have also been found to respond to drought tolerance [39]. In this study, we identified several genes related to stress; *MdSOS1*, *MdSOS2*, *MdSOS3*, *MdWRKY30*, *MdAREB1A*, *MdAREB1*, *MdRD29A*, *MdRD29B*, and *MdNHX1* exhibited significant upregulation trends in high-salt-resistance rootstocks (Figure 7), indicating that they have strong responsiveness to their related genes under salt stress and can quickly transmit salt stress signals to better resist the effects of salt stress. Their molecular mechanisms need to be further investigated. In addition, six genotypes of rootstock hormones underwent varying degrees of changes under salt stress (Figure 6). ABA is one of the important endogenous hormones in plants. Under salt stress, plants can accumulate ABA [40], resulting in cell closure and the expression of related genes [41], thereby enhancing their ability to resist stress [42]. Consistent with our research, the ABA content in the high-salt-resistance rootstock increased under salt stress, and hormones can regulate plant growth and development, as well as plant responses to stress [43]. Stress response hormones include abscisic acid (ABA), gibberellin 4 (GA_4_), salicylic acid (SA), etc. According to reports, plants with strong adaptability to salt stress environments reduce the negative effects of salt stress by regulating endogenous hormones, thereby maintaining growth balance [13]. Among them, SA, as an inducing substance, mainly regulates membrane lipid peroxidation by increasing SOD and POD activities, resulting in a significant increase in chlorophyll content and photosynthetic rate. Consistency with the antioxidant enzyme trends in the previous section of this study (Figure 5) further confirms our position, but the GA_4_ change trends were not obvious. We confirm that high-salt-resistance rootstocks can reduce the harm of salt ions by regulating the endogenous hormones themselves, which provides some reference for future research on the mechanism of endogenous hormone changes under salt stress. In summary, this study indicates that plants exhibiting high salt resistance can adapt well to salt stress environments, highlighting their key role in the future development of germplasm resource production. Overall, this high-salt-resistance apple rootstock is a valuable germplasm resource for cultivation.

In this study, the physiological indicators of high-resistance ‘71-3-150’ and ‘54-118’ remained in a positive state under adversity compared to the control group. The genotypes of different apple rootstocks are different. High-resistance rootstock may better maintain ion homeostasis under the influence of salt stress [3], actively resist osmotic stress through the mutual balance between ions, and have less leaf damage and stronger photosynthesis. Their endogenous synthesis of small molecule compounds jointly balances salt stress harm. Among them, we believe that the stress resistance pathway of high-resistance rootstocks plays a crucial role in activating the plant antioxidant enzyme system, which can better regulate and balance hormone contents in the plant and activate the transcription and high expression of salt-related genes more quickly. On the contrary, low-salt-resistance rootstocks ‘ML176’ and ‘ML2’ break the ion balance under the influence of adversity, resulting in ion toxicity. The accumulation of Na^+^ in the plant body leads to competition between ions, resulting in a significant decrease in the content of various indicators such as fresh weight, chlorophyll, and water content under salt stress (Figure 2). Simultaneously, we believe that low-salt-resistance rootstocks have low antioxidant enzyme activity, which hinders endogenous hormone synthesis in the body, and plants cannot induce the relevant salt-resistance genes to resist the effects of salt stress. Overall, high-salt-resistance apple rootstocks have the potential for the development and utilization of salt-resistant germplasm resources in the future.

## 4. Materials and Methods

### 4.1. Plant Materials

The apple rootstock materials are as follows: asexual tissue apple rootstock and rooted apple rootstock ‘71-3-150’ (Russia), ‘54-118’ (Russia), ‘M9T337’ (The Netherlands), ‘GM256’ (Jilin Academy of Agricultural Sciences Fruit Research Institute, Jilin, China), ‘ML176’ (Shandong Institute of Pomology, Tai’an, China), and ‘ML2’ (Shandong Institute of Pomology, Tai’an, China).

### 4.2. Experimental Design

The apple rootstocks were obtained from the Taiping Lake Experimental Base of the Shandong Institute of Pomology. Apple rootstock types with similar growth stages were selected for control and salt stress treatments. Six types of rootstocks (‘71-3-150’, ‘54-118’, ‘M9T337’, ‘GM256’, ‘ML176’, and ‘ML2’) were selected for experimental pretreatment. In total, 180 bottles of apple rootstocks (six rootstocks were treated with control and salt stress, respectively; 50 rootstocks of each type; 25 for control and 25 for salt stress; the control and salt stress treatments were repeated 3 times) were cultured in the culture medium. The control group was cultured in one type of medium (MS + 0.5 mg·L^−1^ 6-BA + 0.5 mg·L^−1^ IBA + 30 g·L^−1^ sucrose), while the salt stress treatment group was cultured in a 150 mM NaCl salt treatment medium [44]. These media were left for more than 20 days. The rooting treatment consisted of 180 bottles of apple rootstock (six rootstocks were treated with control and salt stress, respectively; 50 rootstocks of each type; 25 for control and 25 for salt stress; the control and salt stress treatments were repeated 3 times) in 1/2 MS + 0.3 mg·L^−1^ IBA + 20 g·L^−1^ sucrose medium. After rooting, the rooting rootstock was transferred to a plastic bowl for cultivation, and the treatment group was grown in a plastic bowl containing 150 mM NaCl for more than 40 days. The physiological indicators and related gene expression responses of several rootstocks in the control group and salt treatment group were examined.

### 4.3. Determination of Growth Indicators

A total of 108 rootstocks were measured for plant height, fresh weight, and the number of leaves. A total of 3 rootstocks were selected for each of the two treatments, and 6 rootstocks were selected for each type, with measurements repeated 3 times. A total of 108 rootstocks were measured for their leaf area and fresh leaf weight. A total of 3 leaves were selected for 2 treatments, and 6 leaves were selected for each rootstock. The measurements were repeated 3 times.

Plant height: the distance from the base of the plant to the top of the main stem was measured using a tape measure with an accuracy of 1 mm.

Plant fresh weight: accurate weighing was carried out using an electronic balance (Qingdao Juchuang Environmental Protection Group Co., Ltd., Qingdao, China).

Number of leaves: the number of leaves visible to the naked eye was counted.

Leaf area: the leaf area was scanned and calculated using Image Pro Plus v.1.53.

Leaf fresh weight: the leaves were accurately weighed using an electronic balance.

### 4.4. Determination of Relative Moisture Content of Leaves

According to Xue’s method, the relative moisture content of plant leaves was determined [45]. Six leaves were cleaned from each of six different apple rootstocks and their fresh weight was measured on an analytical balance (Qingdao Juchuang Environmental Protection Group Co., Ltd., Qingdao, China), denoted as Wf. Then, the leaves were immersed in pure water for 24 h and wiped clean; their saturated fresh weight was measured and denoted as Wt. Then, immediately after weighing, they were boiled at 105 °C for half an hour. They were then dried at 80 °C until they reached a constant weight. Finally, their dry weight was measured and denoted as Wd.

### 4.5. Determination of Malondialdehyde

Using the thiobarbituric acid method, 0.5 g of plant leaf tissue was weighed and cut; then, 2 mL of phosphate buffer solution was added and ground evenly. Finally, 5 mL of 0.5% thiobarbituric acid solution was added and mixed well, and the test tube was boiled in water for 10 min. After the test tube cooled, the centrifuge speed was set to 1200 r·min^−1^, and the mixture was centrifuged for 5 min. Then, 2 mL of the phosphate buffer solution was used, and 5 mL of 0.5% thiobarbituric acid was used as the control. The spectrophotometric values of the supernatant were measured at 450 nm, 532 nm, and 600 nm [46].

### 4.6. Determination of Relative Conductivity

Using Chen’s method, the plants were rinsed three times with water and cut into long strips; then, samples of the same mass were obtained. These steps were repeated three times, and 0.2–0.5 g was placed in 15 mL centrifuge tubes. Then, 10 mL of deionized water was added, and the sample was soaked at room temperature for 2 h. Conductivity R_1_ was measured, and the tube was boiled in water for 30 min before being cooled to room temperature. Then, conductivity R_2_ was measured [46]. The calculation formula is as follows:

$$Relative\;conductivity\left(\%\right)=\frac{R_{1}}{R_{2}}\times 100\%$$

### 4.7. Observation of Leaf Tissue Staining

DAB and NBT staining [47]: Before the experiment, 1 mg·mL^−1^ DAB staining solution was prepared. The pH was adjusted to 3.8 with NaCl (freshly prepared), and a 0.5 mg·mL^−1^ NBT staining solution was also freshly prepared. Then, the staining solution was divided into triangular bottles, and the pretreated plant leaves (a total of 180 rootstock leaves; 5 for each of the two treatments, 10 for each type, repeated 3 times) were added to the bottles. A vacuum pump was used to extract air, and the samples were stained at 28 °C in the dark for 8 h. They were checked at 5 h to avoid excessive staining; then, the staining solution was discarded, and the samples were boiled in a fixative (ethanol:lactic acid:glycerol = 3:1:1) for 5 min until all chlorophyll was removed. Finally, the sample was cooled, and anhydrous ethanol was added.

### 4.8. Determination of Chlorophyll

Leaves with uniform growth were taken from the same position to avoid the main vein, and leaf fragments were randomly selected. The two treatments were weighed at 0.05 g each, and this process was repeated three times. Then, 95% ethanol was added, and the samples were soaked overnight at room temperature in the dark. The samples were shaken multiple times. If all plant materials turned white, chlorophyll was completely extracted (photos were taken with the dye). A spectrophotometer (Shimadzu (Suzhou) Instruments Manufacturing Co., Ltd., Suzhou, China) was used to measure the absorbance values of the extraction solution at 649 nm and 665 nm, and the total chlorophyll, chlorophyll a, and chlorophyll b contents were calculated based on absorbance [48].

### 4.9. Determination of Anthocyanins

Apple rootstock leaf tissue was soaked in hydrochloric acid methanol solution and extracted overnight at 4 °C in the dark. Subsequently, the anthocyanin extract was obtained by centrifugation at 4 °C and 12,000 rpm for 15 min. Then, the absorbance of the anthocyanin extracts was measured at 530, 620, and 650 nm using a spectrophotometer (UV 8000S, METASH, Shanghai, China). Anthocyanin contents were calculated using the formula (A530 − A620) − (0.1 × (A650 − A620)) [48].

### 4.10. Observation of Plant Tissue Slices

Staining was carried out with 0.05% toluidine blue (TB), and the stained leaf tissue was observed under a microscope [49].

### 4.11. Determination of Superoxide Anions and Hydrogen Peroxide

The NBT assay kit was used, following the manufacturer’s instructions (Keming, Suzhou, China).

### 4.12. Determination of Ion Content

Experimental plant materials were heated at 105 °C for 0.5 h; then, the sample was dried at 65 °C to a constant weight (approximately 2 days). The samples were ground into powder and placed in a 50 mL conical flask. Next, 5 mL of concentrated sulfuric acid was added overnight (fume hood). The sample was covered with a curved neck funnel (diameter: 4 cm) and simmered at 220 °C When a small amount of bubbles appeared in the liquid and white mist appeared in the triangular flask, a small amount of 30% H_2_O_2_ was added dropwise; then, more was slowly added until the solution color became clear. A small amount of ddH_2_O was added to the boiled liquid. Then, the materials were shaken well and left to cool before being filtered through 2 layers of filter paper into a 50 mL centrifuge tube with ddH_2_O. The conical flask was rinsed. Finally, the volume was adjusted to 40–50 mL. Then, the corresponding calibration curves were obtained based on the measured elements using a flame spectrophotometer (Shanghai Hongji Instrument Equipment Co., Ltd., Shanghai, China) [50].

### 4.13. Determination of Oxidase Activity

The activities of SOD, POD, and CAT in the leaves were measured using a micro-detection kit and a UV spectrophotometer, in accordance with operating instructions (Keming, Suzhou, China).

### 4.14. Determination of Penetrating Substances

The contents of proline, soluble sugar, and soluble protein were obtained as follows: a micro-detection kit was used, following the operating instructions provided by the manufacturer (Keming, Suzhou, China).

### 4.15. Determination of Endogenous Hormones

The contents of abscisic acid (ABA), gibberellin acid 4 (GA_4_), and salicylic acid (SA) were determined using liquid chromatography–mass spectrometry (LC-MS/MS). Based on the Sciex 4500 LC-MS/MS platform, the content of plant hormones was detected using (http://www.targetcrop.com/ (31 May 2024)). Each experiment was repeated 3 times or more.

### 4.16. Related Gene Expression

The total RNA was extracted from plant tissues using the Mona Biological RNA Kit (Mona Biotechnology Co., Ltd., Suzhou, China, Suzhou Industrial Park). The RNA was transcribed into cDNA, and qRT-PCR quantitative analysis was carried out using a PCR instrument (Suzhou Molarray Co., Ltd., Suzhou, China). MonScriptTM5x RTlll All in One Mix, MonScriptTM dsDNase, and Nuclease Free Water (Jiangsu Cowin Biotech Co., Ltd., Taizhou, China) were used for reverse transcription and qRT-PCR, respectively. The 2^∆∆CT^ method was used to calculate the relative expression levels of selected genes. The reference gene was 18s (Table 1).

### 4.17. Data Analysis

Data for statistical analysis were analyzed using Excel2010, SPSS 22.0, and GraphPad Prism v.8.0.2. Duncan’s multiple mean comparison test was used to distinguish significant differences between treatments based on different letters, with *p* < 0.05. GraphPad Prism v.8.0.2 was used for plotting.

## 5. Conclusions

This study evaluated the adaptability of six rootstock types to salt stress. The experiments demonstrated that high-salt-tolerance apple rootstocks may resist salt stress by regulating the accumulation of reactive oxygen species and endogenous hormones, controlling ion homeostasis, and inducing the expression of related genes more effectively. Based on various indicators of six rootstocks evaluated in existing studies, their salt tolerance is as follows: high-salt-resistance rootstocks ‘71-3-150’ and ‘54-118’, medium-salt-resistance rootstocks ‘M9T337’ and ‘GM256’, and low-salt-resistance rootstocks ‘ML176’ and’ML2’. High-salt-resistance rootstocks will greatly expand the planting area for apples in the future. The apple industry’s quality and efficiency will also significantly improve, increasing the adaptability of apple rootstocks to salt stress and meeting the needs of different environments and regions. High-salt-resistance apple rootstocks can provide favorable support for adaptability and development. Among these, high-salt-tolerance rootstocks ‘71-3-150’, and ‘54-118’ can serve as important germplasm resources for cultivating salt-tolerant apple rootstocks. Apple rootstocks with high salt tolerance can be used as optimal rootstocks to improve salt stress resistance. In future apple production, it remains to be explored whether the high-salt-tolerance rootstocks ‘71-3-150’ and ‘54-118’ also have good adaptability to drought and other adverse stresses.

## Figures and Tables

**Table 1:** Salt-induced gene sequences.

18s	F:ACACGGGGAGGTAGTGACAA R:CCTCCAATGGATCCTCGTTA
MdSOS1	F:TCCGGTTAATCCATCACACACCGT R:TTTGCTGCCCTGGAGGATTTGTTG
MdSOS2	F:TTAGTGGACAGGGTTACGA R:CCATTGAGTTCGCTACAGC
MdSOS3	F:GGTGTGAATGTGAAGATGAT R:CACAACTGACTCGACG
MdAREB1A	F:CAGAGAATCAGCTGCCAGGT R:TCTCCATGTCCTGATTCTTC
MdAREB1B	F:TTAGAACTAGAGGCAGAAGT R:CTGTCAATGTTCGTCGTAAG
MdWRKY30	F:AAGGGAGGGATCTGGCTAGG R:TGAGGCTGAGCTGCTAGAGT
MdRD29A	F:CTGAAGAAGGTAAAGGAGAA R:CCTTCAAAATATCTCCTTGC
MdRD29B	F:CCAAATTACCATGCCTCAAC R:CCTTGGACTTGTACTCTCCC
MdNHX1	F:CATCGCTATTGGCGTAAGT R:TTGTTCCGTTCAGTTGGTG

## Data Availability

Data will be made available upon request.

## Abbreviations

ABA	Abscisic acid
GA_4_	Gibberellic acid
SA	Salicylic acid
FW	Fresh weight
ddH_2_O	Double distilled water
DW	Dry weight
FW	Fresh weight
MDA	Malondialdehyde
H_2_O_2_	Hydrogen peroxide
O_2_	Superoxide anion
NBT	Nitro Blue Tetrazolium
DAB	Diaminobenzidine
CAT	Catalase
POD	Peroxidase
SOD	Superoxide dismutase
